# Ethical Environments of the Dental Surgeon

**Published:** 1892-01

**Authors:** Albert Westlake


### ETHICAL ENVIRONMENTS OF THE DENTAL SURGEON.[1]

BY ALBERT WESTLAKE, D.D.S.

While listening to the eloquent discussion on "Dentistry of the Present Day," and kindred topics, at the June meeting of the Central Dental Association of New Jersey, I recalled the substance of a report which I once read in an old English medical journal of a meeting in 1844 of the Royal College of Surgeons.

The chief point of contention was the question, whether the future dentist should be a member of the College of Surgeons?

It was well known that the examiners of the college never put questions that had any relation whatever to dental surgery.

It was contended that the best dentists were not "qualified" men, and that as "drawing teeth" was a mere mechanical operation, a blacksmith was as good a dentist as a surgeon. In fact, one of the prominent surgeons present declared that a dentist with whom he was acquainted had been originally of that calling, and the learned doctor considered that his early education rendered him *more eligible as a dentist than though he had been educated as a surgeon!*

---

[1] Read before the Central Dental Association of Northern New Jersey, December, 1890.

Could our learned doctor of England have been transported over three thousand miles, and the past thirty-five years, his intellectual armor would need finer temper than could have been welded by his "blacksmith dentist" to have withstood the brave onslaught made by Doctors Atkinson, Stockton, Dwinelle, Bonwill, and others, on the obsolete practices of some of the doctors of medicine in the domain of dental surgeons.

It is often demonstrated to the members of the dental profession that there are yet doctors of medicine and surgery who,—

> "Perch'd near *Science'* top,
> Sit only watchful with their heavy wings
> To cuff down new-fledged *talents* that would rise
> To nobler heights, and make the groove harmonious."

It may be said that there is a natural tendency in man's mind to bow to intellectual authority, and there are many to whom intellectual slavery is natural and easy.

A distinguished prelate once remarked that "Authority is the greatest and most irreconcilable enemy to truth and argument that this world ever furnished. All the sophistry, all the color of plausibility, all the argument and cunning of the subtlest disputers in the world, may be laid open and turned to the advantage of that very truth which they designed to hide or to depress, but against authority there is no defence. It was authority that would have prevented all reformation where it is, and which put a barrier against where it is not."

The day of "systems" has practically passed. The love of truth for its own sake and devotion to independent inquiry has placed the dental surgeons to-day where the medical profession, as a whole, is forced to recognize their discoveries and deductions.

This brings us to the "code of ethics," a subject that will be discussed more in the future than it has been in the past.

In years gone by, when one's knowledge was wrapped up in his anatomical frame-work and the four walls of his office, the ethical shoe pinched only when a secret was out. Petty jealousy of the superior reputation or worldly success of another is below the high principles of the nicest honor which should be expected of members of a noble calling.

To-day the door is wide open, and he can pass up and possess the Beulah land of knowledge who spares no toil, who shrinks at no sacrifice of ease or momentary enjoyment, who feels elevated at the grandeur of the end at which he aims, and by his very energy spurns away difficulties which otherwise must thwart and overcome him.

We are in a profession which, like that of medicine, while it summons into active play the strongest powers of the understanding, tends to foster the kindliest feelings of the heart. This is nominally true as regards our painful surgical operations and intercourse with our patients, and it should be so in the field of fraternal fellowships. That our medical brethren are accepting our right of position in the "sphere" of professional life is perforce conclusive.

Those dental surgeons whose practice is confined to the towns and smaller places (and some cities) are often reminded of the vagaries of surgical knowledge. In June a young man, aged twenty-five, came to consult me in regard to a swelling near the angle of the inferior maxilla. He said that Dr. X. (an M.D.) had prescribed an ointment to be rubbed on the affected surface for three days. Finding no improvement, he came to me. On examination, I found an *impacted* "wisdom tooth." Being unable to reach it in the ordinary way, owing to the tension of the muscles, I was obliged to sacrifice the twelfth-year molar, and then reaching with a straight incisor forceps, worked the offending third molar out. The simple remedy of removing the cause to effect a cure.

CASE II.—Miss F., aged approximately twenty-five, came to me about ten months ago, and said that her physician, after one year of treatment, advised her to go to a foreign sanitarium, as she had "heart-disease." Before leaving she desired to have a tooth extracted that annoyed her. Upon looking into her mouth the remark made by one of our college professors, that "ninety per cent. of the so-called cases of heart-disease are due to indigestion," came to me. The statement is broad, but at times valuable from which to make deductions.

I suggested that she should postpone her trip until I could extract *all* the roots on the superior arch, there not being a sound tooth there, and the surrounding tissue being greatly congested. She consented. After extracting, I sprayed a four-per-cent. solution of cocaine, and, dissecting the gums, excised *the alveolus;* then cutting off the ragged edges of gum, I dismissed her with a regimen.

In three weeks I had the temporary case inserted, and to-day not only has the "heavy feeling," as she described it, and the palpitation of heart, after eating, disappeared, but she has gained eighteen pounds in weight.

CASE III.—Lad, aged ten years, complained of pain in the ear; was treated constitutionally by a medical doctor for a week, at the end of which time a swelling appeared under the left eye. The surface was actually poulticed by the physician's direction, and when he deemed the case "ripe" he applied the knife. The pain continuing, the parents brought the child to my office. I found that although the sixth-year molar had apparently been extracted, yet there was indication of a root remaining. By probing I located the root, which had evidently punctured the floor of the antrum. Using a broken-pointed excavator, I succeeded in bringing the offender out.

Enlarging the opening, I washed the parts thoroughly with a warm solution of chloride of sodium. The odor being offensive, I injected diluted permanganate of potash, then full strength peroxide of hydrogen, and at later sittings hydronaphthol and aromatic sulphuric acid, and can report the case cured. I found small portions of necrosed bone, which I removed with suitable trephines. The sight of the left eye was impaired by the floor of the orbit being raised and the optic nerve stretched, and the nose was swollen, all of which plainly indicated abscess of antrum.

Case IV.—Miss B. had seven gold fillings inserted in the spring of 1890, and I dismissed her with the assurance that her teeth were in good condition. No apparent indication of caries in any of them. Returned, after absence of six months, with proximal and fissure cavities in five teeth, and others considerably corroded. On inquiry, learned that she had been under a physician's care for general debility, and had been taking tincture of iron. After again putting her teeth in order I called upon her physician and inquired if he administered the tincture to secure local effect on the throat, the patient being subject to ulcerated sore throat; he said, "No."

"Then why don't you give the remedy in capsules, or ferrum dialysatum in Vichy water through a tube?"

"Oh! I guess what I give won't injure the teeth, and if it does, 'tis good for your business."

Another case was where the same medical doctor was implicated. A young woman called, suffering with an alveolar abscess. Her friends insisted that she call to see me, although her physician had advised her to take some remedies which he gave her and to wait until the swelling had decreased before she had the tooth extracted. I told her in as simple a manner as was demanded that to secure the contents of a bottle we must either remove the cork or, by a slower method, make a hole through it. She finally understood the parable and application and was relieved. She belonged to that class who tell you that her grandfather's teeth were all double and "he finally shook them all out."

The cases cited above are typical ones, and doubtless occur in every-day practice of many dental surgeons; but I have one that is, I think, new, yet it illustrates the idiosyncrasies of some practitioners.

A lady called upon me and said that her physician (a Homœopathist) requested that she should have all the amalgam fillings in her teeth removed and gold inserted, as "the mercury in the filling was injurious and it did not allow his medicine to act."

I found the fillings were evidently put there by an operator who had conscience and skill, and I told her it would be better to go back to catnip tea or a "lower dilutionist" than to disturb such good work.

I only cite these cases to make the point that the medical profession must recognize the fact that they have no more warrant to trespass on our specialty of oral surgery than has the dental surgeon in obstetrics. "Render unto Cæsar that which is Cæsar's." One of the vital considerations of the day, within the power of the family physician to control, is the importance of having the mouth and teeth of pregnant women kept in healthy condition. How much suffering could be prevented to the mother, and what possibilities of amiable temperaments in the offspring, if, when the family physician is cognizant of the pregnancy of the woman, he should advise and insist that she engage the services of a competent dental surgeon. Some high-minded and sympathetic practitioners do anticipate the comfort of their charges in this respect, and refer them to those dental surgeons whose skill and tender manipulations relieve the tension of their patient's hypersensitive nerves.

In this and many other ways the public will be brought to a more intelligent understanding of the scope of our profession. There is power in a name under some circumstances. I love to hear the changes rung on the words dental surgeon. I believe the day will come when the current pseudonyme *dentist* will be applied only to the empirical.

The members of the dental profession are rapidly coming under the canopy of fraternal fellowship, from which we can go forth into the sunshine of knowledge that is now on the rise, and, keeping our intellectual pores open, absorb the unfolding mysteries of physiological chemistry and surgery as applied to our exigencies.

